# Magnesium Potassium Phosphate Cement-Based Derivatives for Construction Use: Experimental Assessment

**DOI:** 10.3390/ma15051896

**Published:** 2022-03-03

**Authors:** Šimon Marušiak, Adéla Kapicová, Adam Pivák, Milena Pavlíková, Zbyšek Pavlík

**Affiliations:** Department of Materials Engineering and Chemistry, Faculty of Civil Engineering, Czech Technical University in Prague, Thákurova 7, 166 29 Prague, Czech Republic; simon.marusiak@fsv.cvut.cz (Š.M.); adela.kapicova@fsv.cvut.cz (A.K.); adam.pivak@fsv.cvut.cz (A.P.); milena.pavlikova@fsv.cvut.cz (M.P.)

**Keywords:** magnesium potassium phosphate cement, setting retarder, lightweight aggregate, rheology, mechanical and physical parameters

## Abstract

The presented research is focused on the development and testing of the magnesium potassium phosphate cement-based materials (MKPC-based). Firstly, the fresh state properties of the pastes consisting of dead burned magnesia powder, potassium dihydrogen phosphate, setting retarder borax applied in the range of 0–10 wt.%, and batch water were investigated. The aim of testing was to characterize the hydration process in dependence on the borax content. The properties of raw MgO powder were described by chemical composition and particle size distribution. The properties tested in fresh state included shear stress (viscosity), Young’s modulus of elasticity, and temperature; their time dependence was observed. The measurements started immediately after the mixing process. At the age of 14 days, basic structural and mechanical properties of the hardened pastes were obtained. The mixture with 5 wt.% of borax proved to be the most advantageous in terms of setting time, sample integrity, and mechanical strength; therefore, it was chosen as the binder for the following part of the study—MKPC-based mortar development. In the next step, the MKPC paste containing 5 wt.% of borax was supplemented by silica sand aggregate, and the resulting material was marked as a reference. Subsequently, three other mixtures were derived by replacing 100% of quartz sand by lightweight aggregate; namely by expanded glass aggregate, waste rubber from tires, and combination of both in ratio 1:1. The aggregates were characterized by chemical composition (except for the rubber granulate), and loose and compacted powder density. For the resulting hardened composites, basic structural, hygric, strength, and thermal parameters were investigated. The use of lightweight aggregates brought in a considerable decrease in heat transport parameters and low water permeability while maintaining sufficient strength. The favorable obtained material properties are underscored by the fact that magnesia-phosphate is considered to be a low-carbon binder. The combination of magnesia-phosphate binder and recycled aggregate provides a satisfying, environmentally friendly, and thermally efficient alternative to traditional Portland cement-based materials.

## 1. Introduction

The research of new environmentally friendly advanced materials that could serve as a replacement for some commonly used materials keeps gaining in importance, as it is one of the ways to improve energy efficiency of the building and construction sector and to lower its CO_2_ production. Decarbonization of the construction sector is closely related to the cement replacement, as the massive global cement production consumes an excessive amount of energy and is responsible for approximately 7% of the worldwide CO_2_ emissions [1,2]. To ensure that the reduction of building emissions by 2050 will reach 95–100% below the 2015 levels and therefore fulfill the targets defined by the Paris Agreement [3,4], there is an effort to replace concrete and mortar in structures by more sustainable and energy efficient materials such as wood, and to replace cement in general either partially by supplementary cementitious materials, or fully by alternative binders [5,6,7,8,9]. Nowadays, various cement replacements are being quite successfully developed; however, their scope of usage is usually limited by their properties and also their availability. Such green material that could completely replace cement in terms of performance, quality, quantity, and cost, has not yet been found [10,11]. Therefore, the search for new, advantageous materials continues.

Among the alternative binders, magnesium phosphate cements (MPCs) are the matter of interest in this research, as their low CO_2_ emissions and advantageous material properties mark them as one of the possible future green substitutes for Portland cement [12]. There are several types of MPCs—some long known, such as magnesium potassium phosphate cement (MKPC) and magnesium ammonium phosphate cement (MAPC), and some newly developed, such as magnesium silicon potassium phosphate cement (MSPPC) [13]. In the past, mainly the MAPC had been widely researched and used, e.g., as a pavement and bridge deck crack repairing material for its fast setting even when subjected to low temperatures, for early strength gain, and for excellent mechanical resistance; moreover, for similar reasons, its possible biomedical use was suggested [14,15,16,17,18]. However, it’s possible that a negative environmental and health impact caused by the released ammonia was discovered, and afterwards, researchers turned their attention to the MKPC, a material that avoids this harmful influence and also enables slowing down the hydration reaction [12,19,20]. Nowadays, MKPC-based materials persist in the center of researchers’ attention as there are still some performance flaws to overcome.

The MKPC, magnesium phosphate salt also called chemically bonded phosphate ceramics (CBPC), is formed by the acid–base reaction of dead burned MgO and potassium dihydrogen phosphate. The reaction product MgKPO_4_∙6H_2_O, also known as struvite-K, provides the mechanical properties to MKPC [21,22]. The hydration process of MKPC and also its final properties are affected by different factors, such as the chosen magnesia to phosphate ratio (M/P), water to binder ratio (W/B), and also sand (aggregate) to binder ratio (S/B) in the case of MKPC-based mortars. The impact of the mentioned factors has been thoroughly researched by various studies [23,24,25,26].

Although a simple increase in the W/B ratio might elongate the setting time to an acceptable value, it is not an option as it negatively affects the resulting material strength [27]. Therefore, another way to control the rapid exothermic reaction while maintaining reasonable strength and also high early strength is needed. Initially, various additives usually serving as cement retarders such as sugar and starch used to be incorporated in MKPCs; however, their effect was minimal [15]. Later on, several approaches were closely examined—the use of magnesia with lower reactivity, the use of retarders, the replacement of potassium dihydrogen phosphate by dipotassium hydrogen phosphate, etc. [26,27,28,29,30]. As for retarders, various admixtures with a possible retarding effect were examined for MPCs, e.g., fly ash [21,31,32], granulated blast-furnace slag [33], alumina cement [31], sodium triphosphate (STP) [34], boron compounds [15,35], composite retarders [36], etc. Of the mentioned examples, boron compounds are likely the most often used ones, as the retardation effect and impact on strength provided by the other possibilities is more complex and not always positive [17,34]. Among boron compounds, borax [17,26,30,31,37,38,39] and boric acid [35,40,41] are usually the used ones, both providing similar retarding effect when used in MPC-based composites [34]. The aforementioned reports indicate that the use of borax and its dosage significantly affects various material properties, some in a good way and some in an unwanted way. Therefore, a proper borax dosage for specific uses must be carefully chosen to preserve all the desired resulting material properties, as no strict limit for borax amount in MKPC mixture can be universally prescribed.

Nowadays, the MKPC can be used as a material for repair works because of its high bonding capacity, rapid setting, and high early strength [42]; it can also be used in the form of possible waste forms immobilizing and encapsulating galvanic waste and radioactive waste [43,44,45]. Moreover, MKPC can be used as a steel protective material for its corrosion and fire resistance [36,46]. Unfortunately, because of the short setting time, some applications such as the MKPC use in the form of ready-mixed composite and its use in high temperatures are not possible. The insufficiently low pH of the MgO-based binders also does not allow the use of the traditional steel reinforcement, and therefore these binders cannot be used in structures subjected to significant tensile stress. These are some of the problems to overcome if MKPC is supposed to become a cement substitute, and the choice of the right retardant and its correct dosage might be the key to success.

As for the thermal properties of MKPC, those are widely researched for several reasons. Firstly, the current trend in material engineering is to enhance thermal properties of building materials and ideally to minimize the heat losses and energy losses in general [47]. Secondly, and more specifically, the MKPC has a potential in the field of fire protection—it has the potential to take over cement’s role in the given field. For those reasons, new advantageous lightweight materials with advanced fire resistance, thermal properties, and the target to be cheap, non-toxic, and also sustainable, are being developed [48,49,50,51,52].

As finding the ideal setting time and improving the mechanical and thermal properties, etc. can widen the possible use of MKPC-based composites, the goal of the presented research was to identify the optimum dosage of setting retarder in composition of MKPC which served as basis for design and development of advanced MKPC composite mortars. In MKPC mortars, expanded glass granules and/or crumb tire rubber were used as full substitutes of silica sand. Both applied aggregates were chosen as they are not only lightweight, but also sustainable (“green”) as waste glass and was crumb rubber are turned back into production process and thus transformed to profitable materials. The use of both crumb rubber and expanded glass is supported by the previous research focused on the use of lightweight aggregates in a different group of magnesia-based cements, specifically magnesium oxychloride cement [53]. The topic of lightweight aggregates was pursued, as for MKPC-based composites this problematic has not yet been properly researched and above all the combination of MKPC and higher volume of various lightweight aggregates still needs to be thoroughly examined. For the prepared lightened MKPC mortars, structural, mechanical, hygric, and thermal parameters were accessed in order to provide a complex view of MKPC properties and hygrothermal performance. To the best of the authors’ knowledge, thermal and hygric parameters of MKPC-based materials were only scantly studied up to now, and the acquired technical characteristics thus represent new and valuable information in the field of development of magnesia-based materials.

## 2. Materials and Methods

### 2.1. Materials and Design of Pastes

The first part of the research consists of the design and testing of magnesium potassium phosphate pastes produced from magnesia oxide (MgO), potassium dihydrogen phosphate (KH_2_PO_4_), tap water, and a setting retarder borax (Na_2_B_4_O_7_·10H_2_O). Both KH_2_PO_4_ and borax in p. a. purity were provided by Lach-Ner s. r. o. Magnesia oxide was produced by SMZ s.r.o. Jelšava, Slovakia. Its chemical composition was assessed by X-ray fluorescence analysis (EDXRF Spectrometer, ARL QUANT’X, Thermo Scientific); the measurement was conducted in the argon atmosphere. Oxide composition was acquired by recalculation of the concentrations by the Uni Quant software. Dry loose density was identified on a gravimetric principle—sample volume was measured in a graduated cylinder, and sample mass was determined using precise laboratory scales. The specific density of the MgO powder was measured by the helium pycnometer Pycnomatic ATC (Thermo Scientific), the expanded combined uncertainty of the test was 1.2%. Specific surface was characterized by the Blaine apparatus. Particle size distribution was determined by a laser diffraction analysis (Analysette 22 Micro Tech plus, Fritsch). Obtained data are summarized in Table 1 and Table 2.

Five types of the MKPC pastes and mortars were prepared; this chapter focuses specifically on the pastes. Based on the previous research [54], the magnesia/phosphate molar ratio (M/P) was chosen equal to 8.0 in order to obtain optimal properties of the resulting material. This ratio remained constant in all prepared mixtures. The value of water/cement ratio (W/C) was set as 0.25 for both pastes and mortars with the intention to reach such spread values of the reference mortar (without borax) that would be in agreement with the European standard EN 1015-2 [55]. As the mortar composition was derived from the paste composition, the W/C value was maintained 0.25 even for the pastes. The correctness of the chosen W/C ratio and its impact on the spread value was verified on one experimental MKPC-based mortar mixture prepared ahead of the main experiment; the spread test was performed in accordance with EN 1015-3 [56]. This value of W/C ratio has also been successfully used in a previous study focused on the MKPC mortars with incorporated biomass ash [57].

For the prolongation of setting time, five different weight ratios of borax (the setting retarder) to binder were chosen in the range from 0% to 10%. The paste mixtures were named based on the incorporated borax amount, with P marking them as paste mixtures—to set an example, MP-5-P is the MKPC paste containing 5 wt.% of borax. The composition of all paste mixtures is presented in Table 3.

At the beginning of the preparation process, raw dead burned magnesia oxide was crushed into fine powder. Then, the dry compounds (MgO, KH_2_PO_4_, and borax) were inserted into the mixing bowl and the stirring process was started; a planetary-type mixer (ELE International) was used. After 1 min, the batch water was added. The mixing continued for 1 min at low speed (paddle 140 rpm and mixing head 62 rpm). Subsequently, the mixing was stopped, the mixer head and the bowl sides were cleaned, and afterwards, the fresh mixture was hand-mixed for 1 min. The final 1 min long mixing was again performed by the laboratory mixer, this time at high speed (paddle 285 rpm, mixing head 125 rpm). The homogenous fresh mixture was then casted into four types of molds. The material casted into a cubic mold (edge length 70 mm) was used for rheological parameter testing; the material in two conical molds belonging to the Vicasonic apparatus and the Vicat apparatus was used to measure dynamic modulus of elasticity and temperature, and the setting properties, respectively. The rest of the fresh mixture was casted into the prismatic steel molds with the dimensions of 40 mm × 40 mm × 160 mm. The prismatic samples were left uncovered at (23 ± 2) °C and relative humidity (40 ± 5)% for 24 h; afterwards, they were demolded and air-cured in the same conditions for the remaining time of hardening.

### 2.2. Materials and Design of Mortars

The second part of the research is aimed at the design and testing of magnesium potassium phosphate-based mortars produced from MgO, KH_2_PO_4_, batch water, borax), and different types of aggregates—silica sand, crumb rubber, and granules from expanded glass (Liaver). The previously mentioned characteristics of MgO, KH_2_PO_4_, and Na_2_B_4_O_7_·10H_2_O also apply to this section. As for the aggregate properties, those are closely described in the following paragraphs.

The composition of mortars is based on the previously introduced MKPC pastes. As mentioned above, the M/P ratio was set to 8.0 and the W/C ratio was set to 0.25. The amount of borax was chosen based on the observed properties of MKPC pastes with different borax dosage; it was set as 5 wt.% of the used binder (except for the case of reference mortar MP-REF-0 without borax), as it was concluded that such dosage provides an adequate extension of the setting time while maintaining a decent material homogeneity and integrity (for details see the results section).

As for the used aggregates, the types were chosen with respect to the current efforts to reduce the energy consumption of the building and construction sector and to the effort to reuse waste material. Expanded glass granulate represents the energy reducing material, as it is an insulation material supposedly providing thermally advanced properties to materials; expanded glass Liaver (L) was used in samples MP-L and MP-L+R. The reused waste material is represented by crumb rubber (R) obtained from waste tires; it was used in samples MP-L+R and MP-R, respectively. The reference samples MP-REF 0 and MP-REF 5 contained silica sand aggregate only, where 5 wt.% of borax was used as a setting retarder.

The used silica sand was provided by Filtrační písky, spol. s.r.o. (Chlum u Doks, Czech Republic); three different fractions (0.063–0.5 mm, 0.5–1.0 mm, and 1.0–2.0 mm) were used. The fractions were mixed together in the mass ratio 1:1:1 to obtain the optimal particle size distribution. The chemical composition of the silica sand was investigated by the X-ray fluorescence analysis (EDXRF Spectrometer, ARL QUANT’X, Thermo Scientific) and the Uni Quant software (Thermo Scientific, Milan, Italy) was used for the data collection and analysis.

The expanded glass granulate (commercial name Liaver) was produced by Liaver GmbH and Co. KG, Ilmenau, Germany. The chemical composition of the aggregate was taken from the technical data sheet provided by the producer, and it is presented in Table 4. In order to obtain the particle size distribution comparable to the one of silica sand, the following four fractions of expanded glass granulate had to be used in the given mixing ratio: 0.1–0.3 mm (23%), 0.25–0.5 mm (22%), 0.5–1 mm (25%), and 1–2 mm (30%).

The rubber aggregate was produced by Montstav CZ s.r.o. (Dolní Rychnov, Czech Republic). The granules were made of mechanically processed waste tires (for details see [58]). The following three fractions were used: 0.063–0.5 mm, 0.5–1.0 mm, and 1.0–2.0 mm. They were mixed together in the mass ratio 1:1:1. The X-ray fluorescence analysis determined only 35 wt.% of elements present in the material, namely 18 wt.% of Zn, 2 wt.% both Ca and Si, 3 wt.% of Fe, and 9 wt.% of S variety. The rest is mostly formed by C, H, and O elements which cannot be detected by this analysis.

**Figure 1:**
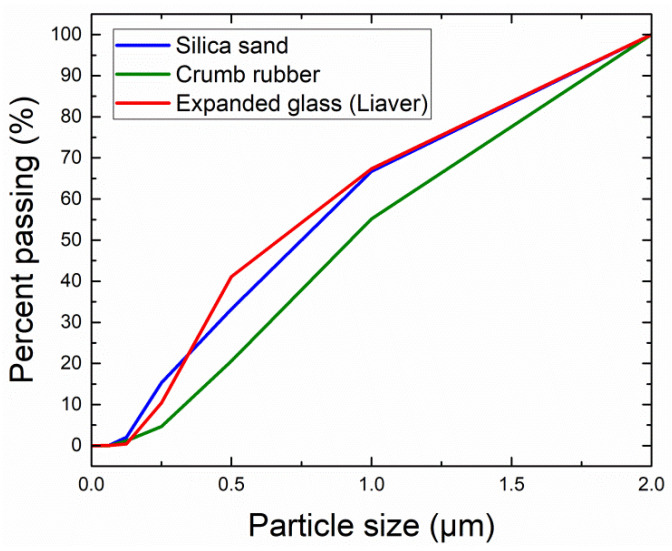
Grading curves of the applied aggregates.

The loose and compacted powder densities of the used aggregates were assessed based on the gravimetric principle briefly described in the previous chapter. Measured data are summarized in Table 4. The grain-size curve of the applied aggregates is depicted in Figure 1.

In this research, there were two reference mortar mixtures prepared, namely MP-REF 0 and MP-REF 5, both consisting of magnesium potassium phosphate binder and silica sand aggregate in the weight solid/binder (S/B) ratio 1.0, where B is actually the active ingredient of the binder—i.e., 76.5 wt.% of MgO + 100 wt.% of KH_2_PO_4_. As mentioned above, the water/cement (W/C) ratio was set to 0.25 in order to obtain the value of spread equal to 165 ± 5 mm (measured on MP-REF 0 before the primary experiment), and the magnesia/phosphate (M/P) ratio of 8.0 stayed fixed. In the case of MP-REF 0, no borax was used, while in the case of MP-REF 5, the borax addition (5 wt.%) was present.

Three different mixtures were derived from the reference mortar MP-REF 5 by volume substitution 100% of silica sand. The first one consisted of magnesia-phosphate binder with expanded glass granulate (MP-L). The second mixture was made by using rubber granulate (MP-R). The last mixture was established by merging both previous aggregates (expanded glass and rubber granulate) in the volumetric ratio 1:1 (MP-L+R). The exact proportions of the prepared mortars are shown in Table 5.

The preparation of the mortar samples was similar to the preparation of the paste mixtures. The only difference was the aggregate addition into the mixing vessel—at the beginning the aggregate was mixed with the other dry compounds. Then, water was added. The order and duration of all the steps of the mixing process previously mentioned in the paste preparation section remained unchanged. At the end of the mixing procedure, fresh mixtures were casted into the prismatic molds with dimension 40 mm × 40 mm × 160 mm. All the samples were left uncovered at (23 ± 2) °C and relative humidity 40 ± 5% for 24 h. Afterwards, the mortar samples were demolded and air-cured in the same conditions for another 13 days with the exception of some of the reference specimens (MP-REF 0 and MP-REF 5) that were subjected to the early strength test at the age 2 and 5 days after mixing. After hardening (at the age of 14 days), various material properties were tested, such as basic structural properties, mechanical parameters, thermal characteristics, and hygric properties. Except for the samples subjected to the strength tests, all samples were dried ahead of testing in the laboratory dryer at 60 °C. Strength tests were performed on the undried samples. The tested material parameters and applied experimental methods are fully described in the next part of this study.

### 2.3. Experimental Methods and Performed Tests—Pastes

The main purpose of the paste manufacturing was to investigate the fresh state material properties of MOC pastes. Additionally, the properties of the hardened samples at the age of 14 days were measured.

The rheological behavior of the pastes was described by shear stress *τ* (Pa) measured by HAAKE Viscometer E (Thermo Scientific). This apparatus belongs into the group of comparative rotation viscometers; it is equipped by a changeable cylindrical rotational spindle (sizes R1–R7). In this case, spindle R7 was chosen with respect to the initial consistency, and the shear rate was set to 80 s^−1^ in order to describe the transition from the fresh to the hardened state in a sufficient detail. The measuring started at the time of approx. 4 min after mixing the dry components with batch water. The test duration was in the interval between 15 and 25 min in dependence on the borax share and therefore on the hardening rate; the measurement was always stopped after reaching maximum measurable value. The obtained shear stress was plotted against time, providing a graph showing the shear stress progress in time.

The evolution of dynamic modulus of elasticity *E_d_* (GPa) and temperature *t* (°C) of the fresh pastes was measured by the Vicasonic apparatus (Schleibinger Ultrasonic Data Logger, Schleibinger Geräte Teubert u. Greim GmbH, Buchbach, Germany), which consists of a measuring cell, two ultrasonic transducers (transmitter and receiver), and a thermocouple. The modulus of elasticity measurement was based on the ultrasonic pulse velocity method and it was evaluated in accordance with the standard ČSN 73 1371 [58]. Both measurements started approximately 5 min after blending the dry components with batch water; their duration time was 14 h.

The setting characteristics described by the initial and final setting time were measured by the Vicat apparatus following the procedure established in the EN 196-3 standard [59]. The time gap between the needle drops was set as 30 or 45 s in dependence on the retarder share. To achieve the best accuracy with the limited number of needle drops, the measurement was run right before the start of material hardening. Test results were obtained in a graphic form and subsequently inserted into the chart.

Basic structural properties of the hardened pastes such as dry bulk density, specific density, and total open porosity, were measured on dried samples; the samples were fully dried at 60 °C before testing. The dry bulk density *ρ_b_* (kg/m^3^) was obtained using a gravimetric principle in accordance with the standard EN 1015-10 [60]; specific density was measured by the automatic helium pycnometer Pycnomatic ATC (Thermo Scientific) in the same way as the aforementioned specific density of MgO. The expanded combined uncertainties of these tests were 1.4% and 1.2%, respectively. The total open porosity *ψ* (%) was calculated as from the obtained bulk density and specific density according to Equation (1)

(1) $$\psi =1-\frac{\rho_{b}}{\rho_{bs}\;}\cdot 100,$$

In the determination of total open porosity, recommendations formulated within the solution of EU project HAMSTAD (Heat Air and Moisture Standards Development) [61] were followed. The expanded combined uncertainty of the porosity assessment was 2.0%.

The compressive strength and flexural strength of the pastes were measured in compliance with the EN 1015-11 [62]. The details on the mechanical test can be found below in Section 2.4.

### 2.4. Experimental Methods and Performed Tests—Mortars

In the case of the mortars, the only examined property of the material in fresh state was the spread diameter. The flow table test was carried out according to the procedure specified in the EN 1015-3 [56] standard. At the age of 14 days, the basic material properties were measured. The tests were performed on dry samples (dried at 60 °C); the testing procedures were identical with the ones performed on pastes that are described in the previous chapter.

Thermal properties of the mortar samples, including thermal conductivity *λ* (W·m^−1^·K^−1^) and volumetric heat capacity *c_v_* (J·m^−3^·K^−1^), were investigated using the thermal constant analyzer Hot Disk TPS 1500 (Hot Disk AB, Göteborg, Sweden) by a nonstationary plane heat source method [63]. The used experimental setup consisted of the Hot Disk apparatus, a sample holder, and a Kapton-insulated sensor serving as the heat source and a temperature sensor at the same time. The sensor was fitted between two halves of the prismatic sample, and the following measurement was based on recording the material response on the thermal energy emitted by the sensor. The samples were dried at 60 °C before testing and measured at the temperature of approx. 20 °C. The expanded combined uncertainties of the thermal conductivity and the volumetric heat capacity tests were both 3.0%.

Hygric properties represented by capillary water absorption coefficient *A_w_* (kg·m^−2^·s^−1/2^), 24 h water absorption *W_a_* (wt.%) and 24 h water absorption *W* (kg·m^−2^) were determined in order to describe the ability of MKPC pastes to absorb and transport liquid water. The water absorption coefficient was determined in the free water intake experiment performed in accordance with the EN 1015-18 [64]. In order to ensure 1D water transport, the examined samples had all lateral sides insulated by epoxy resin. Before the experiment, the samples were dried at 60 °C in accordance with the mentioned standard [64]; afterwards, the samples were cooled down and the measurement was performed at (21 ± 2) °C. During the experiment, the bottom surface of the samples (40 mm × 40 mm) was immersed into distilled water, and the mass increase was automatically monitored for the entire duration of the experiment; the sufficient water amount in the reservoir was periodically controlled to maintain the flawless contact of sample with water. Subsequently, the measured cumulative mass of water was plotted against square root of time, and the capillary water absorption coefficient was calculated as the slope of the initial stage of the previously obtained chart [65,66]. The 24 h water absorption W was measured in compliance with the EN 1015-18 [64]. For the assessment of 24 h water absorption *W*_a_ (wt.%), the prisms cut from the originally casted specimens were used. The prisms had the dimension 40 × 40 × 60 mm^3^. The dry specimens were weighed and freely immersed in water for 24 h. The mass of the wet sample *m*_w_ (kg) was measured and the 24 h water absorption was calculated using following formula:

(2) $$W_{a}=\frac{m_{w}-m_{0}}{m_{0}}\cdot 100,$$

where *m*_0_ (kg) is the dry sample mass.

The expanded combined uncertainty of the tests was 2.3% for the water absorption coefficient, and 1.2% for both 24 h water absorptions.

The mechanical resistance of the mortars was characterized by compressive strength and flexural strength. Both tests were carried out according to the standard EN 1015-11 [62]; for the flexural strength test, the samples with the dimensions 40 mm × 40 mm × 160 mm were used. The compressive strength tests were performed on halves of the prismatic samples previously used for flexural strength tests, with the loading area 40 mm × 40 mm. Both tests were performed on undried samples. For both reference mortars, i.e., MP-REF 0 and MP-REF 5, early strengths at the age of 2 and 5 days, and also the strength values at the age of 14 and 28 days were measured, to observe the strength development in time. As for the samples MP-L, MP-L+R, and MP-R, strengths for those were measured after 14 days only, as the reference samples proved that the difference between the strengths measured after 14 days and 28 days is minimal. The expanded combined uncertainty of both mechanical tests was 1.4%.

The microstructure and morphology of mortar composites at the age 14 days was observed by scanning electron microscope (SEM) Phenom XL Desktop SEM equipped by detector SED and operation energy 5 kV. Samples fractions were viewed at the 1000× magnification.

## 3. Results and Discussion

### 3.1. Properties of the Paste Mixtures

The setting parameters measured by the Vicat apparatus are shown in Table 6. It is obvious from the results that the dosage of borax directly affects the course of the hydration process; the higher the borax dosage, the longer the paste setting time. The shortest initial setting time was observed for MP-0-P (control mixture without a borax additive), where the length of initial setting time was only 9 min, and after another 2 min, the setting was completed. The elongation of setting time was then achieved by the application of borax retarder. By the use of 2.5 wt.% of borax, the setting start was postponed to 11:15 min after mixing dry components with water, and the setting duration was extended to 7:45 min. The use of 5 wt.% of borax addition resulted in the initial setting time being 13 min and the length of the setting period to 9 min; 7.5 wt.% of borax postponed the initial setting time to 14 min, and elongated the setting duration to 10:30 min. The longest setting, 13:30 min, started at the time 17:30, and it was achieved by the mixture MP-10-P with 10 wt.% of borax. Overall, the growing borax share in the developed pastes postponed the setting start by up to 8.5 min, and elongated duration of setting by up to 11.5 min compared to the reference sample; such achievement shows that the use of borax in MKPC pastes allows wider material application assured by the elongated setting time. Gained results exceed the set expectations based on the previously reviewed studies. The reached final setting times are longer than those ones measured by Li et al. [30], who manufactured samples with 5% and 10% of borax (mass percentage of the used MgO) that provided the final setting time 15 min and 25 min, respectively. The obtained results are also more promising than the initial setting times obtained by Wen et al. [39], who measured initial setting times around 2 min, 2.5 min, 2.5 min, and 3.5 min for 2.5%, 5%, 7.5%, and 10% of borax, respectively. However, it should be taken into account that the used amount of borax in the mentioned experiments was slightly lower, as it was set as the mass percentage of MgO, while in this experiment, the borax share was calculated as the mass percentage of MgO + KH_2_PO_4_.

**Figure 2:**
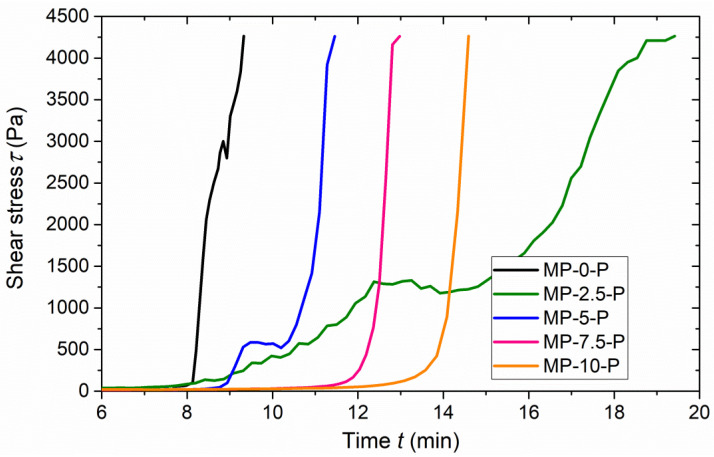
Shear stress development in fresh MKPC pastes.

The results of the rheological behavior measurement, closely connected with the aforementioned setting characteristics, are shown in Figure 2. The performed measurement of the pastes’ shear stress development captures in detail the beginning of the transformation of the fresh mixtures into the hardened material. The presented curves show the progress of the hardening process before reaching the initial setting time; the time of measuring in each case is adjusted to the measuring range of the used apparatus. These results broaden the previously gained findings describing the setting process, and they also describe viscosity of the tested materials.

The pictured curves can be divided into two sections. The first curve section, which can be described as the period before the start of the hardening process, is characterized by a very little growth of shear stress values. Afterwards, the hydration process starts and the slight growth in shear stress changes into the rapid increase; in that period, the initial fluid mixture gradually changes into the semi-solid and later solid composite. With respect to the testing principle, the Viscotester is able to measure the mixtures only in their viscous state. Therefore, the results only provide the information about the course of the hydration process beginning, as the measurement was stopped when the maximum value of the measuring range was reached; the maximum reached shear stress value was the same for all the experiments. Theoretically, it can be expected the progressive increase in shear stress curves until the final setting time, where the curves will reach a plateau.

The curves of all the tested mixtures fully captured the start of the transition from liquid to solid state manifested by the rapid growth in viscosity. For all the cases except MP-2.5-P, similar shape of the steep part of the shear stress curve was detected independently of the borax content; however, it is obvious that the period preceding hardening was elongated based on the used borax amount. In the case of the curve describing the mixture MP-2.5-P, more moderate shear stress growth was observed compared to the other cases. Such result could be caused by reaching some specific binder to retarder ratio associated with a slower onset of hydration reaction. This finding is in agreement with the observed development of temperature and Young’s dynamic modulus (presented below). In both MP-2.5-P and MP-5-P curves there were recorded horizontal steps that were followed by further increase in the shear stress values. However, after reaching approx. 10 min, the increase in shear stress curve was much steeper in the case of M-5-P than recorded for MP-2.5-P. It was similar to those obtained for MP-0-P, MP-7.5-P, and MP-10-P. Therefore, the performance of MP-2.5-P in comparison to other research mixtures can be considered as unique and may lead to the prolongation of the hardening time and retardation of strength development.

As indicated above, the starting moment of the rapid shear stress growth is important, as it marks the start of the hydration process. In the case of the reference mixture MP-0-P (without borax), the onset of hydration was detected at the time 8 min after mixing water with binder. On the other hand, when using 10 wt.% of borax, the time reached 13 min. Using 5 wt.% provided an extension of 1 min compared to the reference mixture. Moreover, the retarder use influenced not only the timing of the hydration, but also the viscosity of fresh mixtures; higher share of borax led to the decrease in viscosity values. It is necessary to note, in the case of MP-5-P, two steep changes in the slope of shear stress curves were identified, first one at approx. 9 min and the second one at 10.2 min. The onset of MP-5-P was attributed to 9 min, the time when the first sharp increase in the shear stress values started.

Overall, the results again demonstrate the effect of retarder use and confirm that the use of borax leads to the time extension of the MKPC hydration process; it specifically shows the elongation of the first (prehydration) period. Moreover, the results reveal that while the borax addition affects the start of setting, it does not affect the shape of the steep part of the shear stress development curve. The results also mostly show (except for the case of the MP-2.5-P mixture) that the transition between the first and the second part of the shear stress curves is milder when using borax; the higher the borax dosage, the milder is the transition.

To avoid any confusion, it is necessary to emphasize that the measurement of viscosity captures the initial period of hardening preceding the initial setting time; the initial setting time occurs approximately 0.5–3 min after the end of viscosity test. Such a fact must be taken into account when setting parameters and shear stress development are compared.

**Figure 3:**
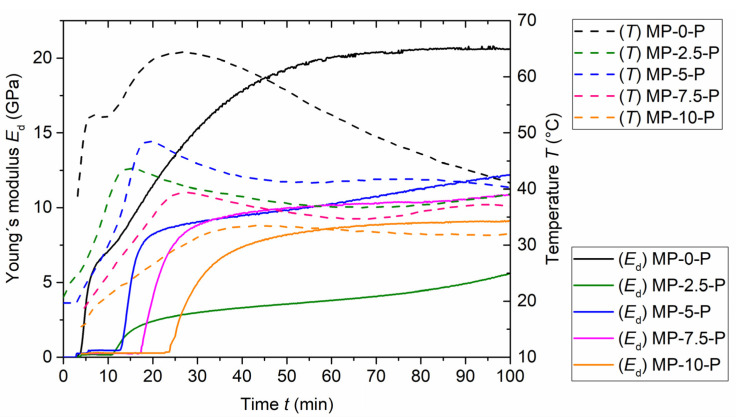
Development of the dynamic Young’s modulus of fresh MKPC pastes, duration 100 min.

**Figure 4:**
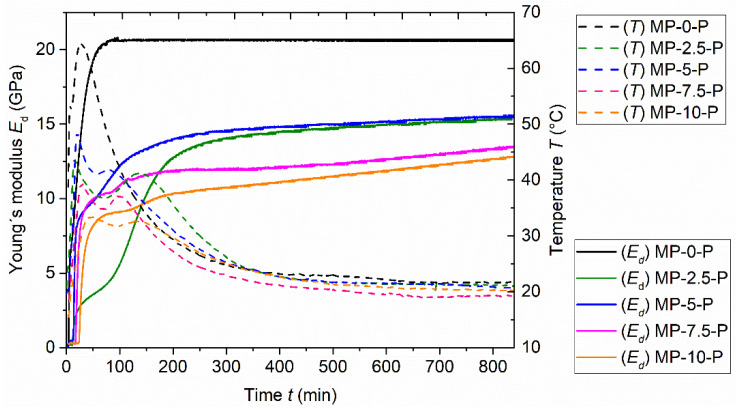
Development of the dynamic Young’s modulus of fresh MKPC pastes, duration 840 min.

The temperature evolution in the fresh paste mixtures is together with Young’s modulus shown in Figure 3 and Figure 4. It is another parameter describing the hydration process, and another possibility to evaluate the efficiency of the borax ratio used in the mixtures. The duration of the temperature change monitoring was for all tested materials 14 h. The values were recorded continuously, with one measured value per 10 s; later on, these values were plotted against time, generating a smooth curve and capturing the temperature changes occurring during the hydration period well.

The results show noticeable differences between the pastes in dependence on borax content. The considerable deceleration of the hydration reaction caused by the increasing borax share is manifested by a slower rate of temperature growth and lower temperature maximum, with this impact becoming more significant with the increase in borax amount. A similar trend of the MKPC paste temperature development in dependence on the borax share was previously observed, e.g., by Yang et al. [37]. The maximum temperature reached, 64.4 °C, was measured for the sample MP-0-P at 26 min after the mixing binder with water. The temperature peaks reached by the mixtures containing borax were lower in all cases, with the lowest peak provided by paste labeled MP-10-P. Similarly, as in the shear stress results, the samples with 2.5% share of borax (MP-2.5-P) showed atypical behavior when compared to the other samples. In this case, using 2.5 wt.% of borax led to the more significant decrease in maximum temperature (43.7 °C) than using 5% of borax (48.4 °C). The use of 7.5% and 10% of borax then led to the expected temperature drop, with the mixture MP-7.5-P reaching the maximum 39.4 °C and the mixture MP-10-P providing the maximum temperature lower than 35 °C.

As for the rate of temperature growth, it might look like that the mixtures MP-2.5-P and MP-5-P reached their maximum temperature earlier than the reference mixture (Figure 2). However, on closer inspection (Figure 3), it is obvious that each temperature curve contains two local maxima—the main peak, and the additional (lower) peak. In the case of the reference sample, the lower maximum occurs first, while in the case of borax-containing samples, the higher maximum occurs first and the lower maximum follows. The first maximum was reached at the time 7.5 min after mixing water with binder by the reference sample. Samples with 2.5%, 5%, 7.5% and 10% of borax reached the first maximum at 15 min, 19.8 min, 27.5 min, and 43.1 min, respectively. It is obvious that the time when the first maximum is reached depends directly on the borax amount—the more borax, the later the peak. Moreover, it is apparent that the borax addition in general affects the course of the temperature vs. time curve.

Generally, borax addition lowered the temperature of hydration reaction in all cases; the highest borax share provided the mildest rate of temperature growth and reached the lowest temperature. It should be mentioned that the most significant differences in the temperature profiles were detected until 100 min after blending water with the binder. After the time of 300 min, the curves remained nearly the same, regardless of the borax ratio.

The dynamic Young’s modulus was measured by the Vicasonic apparatus alongside the temperature profiles. The Young’s modulus change was monitored continuously for 14 h in time intervals 10 s; subsequently, the measured values were plotted against time as shown in Figure 3 and Figure 4. The results are in agreement with the temperature development and the recorded setting times.

The measurement started approximately 4 min after mixing the binder with batch water. In the beginning phase, the values of Young’s modulus were very low and stayed nearly constant until the start of hardening. The initial stage of the hardening process was accompanied by a rapid increase in Young’s modulus values. In the case of the mixture without borax additive, the significant increase was observed from the start of the measurement (4 min). As expected, the mixtures containing borax proved to have a later Young’s modulus increase, with the longest period before increase provided by sample MP-10-P (24 min); according to the results, the time of Young’s modulus rapid increase is directly dependent on the borax amount. However, there is a remarkably small difference between samples MP-2.5-P and MP-5-P—the increase in the case of MP-5-P starts only 2 min later than in the case of MP-2.5-P, while in the case of MP-7.5-P and MP-10-P, the time difference exceeds 5 min, and an even longer time difference was observed between samples MP-0-P and MP-2.5-P. Moreover, the rate of the initial growth of MP-2.5-P elastic modulus values is notably milder; such fact is in accordance with the previous observations.

As for the final Young’s modulus values, the MP-0-P reached the final dynamic modulus (20.6 GPa) quite quickly—approximately 100 min after mixing; afterwards, it remained approximately constant until the end of the measurement. On the contrary, in the case of samples containing borax, the modulus values kept growing until the end of the measurement. The growth was rapid at first, and after 15–42 min (in dependence on the borax share), it became more moderate. The measured modulus values of the samples containing borax were overall significantly lower than those measured for the reference sample. At the age of 100 min, the values of the samples with borax reached approximately 50% of the MP-0-P Young’s modulus value. After 840 min, the values gained for mixtures with 2.5 wt.% and 5 wt.% of borax were both approximately equal to 15.5 GPa. The difference between MP-7.5-P and MP-10-P was also negligible; their Young’s modulus reached approximately 13 GPa.

The gained results show that by adding borax, the dynamic elastic modulus development is postponed, slowed down, and also elongated. Moreover, in the case of sample MP-2.5-P, a significantly milder curve increase can be observed. The values for all borax containing samples keep growing until the end of the measurement. Overall, the results of the dynamic elastic modulus time development are in agreement with the aforementioned temperature development. The rapid increase in temperature is accompanied by the significant dynamic elastic modulus growth. In Figure 4, it can be observed that the duration of the rapid Young’s modulus growth corresponds with the time necessary to reach the first temperature peak, especially in the case of samples MP-5-P, MP-7.5-P, and MP-10-P. Moreover, it should be noted that in both cases, the irregular behavior of the samples containing 2.5% of borax can be observed in the form of slower growth of values and also lower and later peaks. As for the comparison with the setting characteristics, the initial setting time that indicates the start of hardening corresponds well with the start of dynamic elastic modulus growth. The slight deviations may be caused by the experiment setup and the laboratory conditions (temperature and relative humidity).

The basic structural properties, namely bulk density, specific density, and total open porosity were measured on fully dried hardened samples; the obtained results are summarized in Table 7. It can be seen that the borax influence on these properties is very little in most cases. The values of bulk density were approximately equal to 2000 kg·m^−3^, the measured values or specific density were close to 3000 kg·m^−3^, and the total open porosity was approximately 35% in most cases. Only sample MP-5-P provided considerably higher bulk density, 2179 kg·m^−3^, and consequently a lower total open porosity, 26%. This specific borax dosage enabled to uniformly distribute paste components which led to the optimum workability of fresh paste and thus formation of more dense hydrated products. The highest value of specific density, 3103 kg·m^−3^, was obtained for MP 0-P.

The addition of borax reduced both examined mechanical parameters. The lowest mechanical strength yielded MP-10-P, paste with the highest borax dosage. On the contrary, MP-5-P exhibited a moderate drop in mechanical parameters only what was compensated by prolonged workability. In this case, the drop in compressive strength was ~9.8% and ~7.6% for flexural strength, respectively.

The part of the research presented above includes the design and testing of MKPC paste mixtures with the intention to choose an ideal share of borax for the use in MKPC-based mortars. Except for the basic structural and mechanical properties, the testing was focused on determining properties of the fresh mixtures. Overall, the gained results show that the use of a varying share of borax retarder causes significant material changes. The increase in borax content caused the elongation of the hardening period demonstrated by longer initial setting time, changes in viscosity trends, and a slower rise in hydration temperature together with lower temperature peaks. The obtained values of Young’s modulus in time only prove the borax’s impact on setting. Thus, it may seem to be obvious that the highest borax share is the most advantageous one, providing the slowest setting and the lowest temperatures reached within the material at the same time.

**Figure 5:**
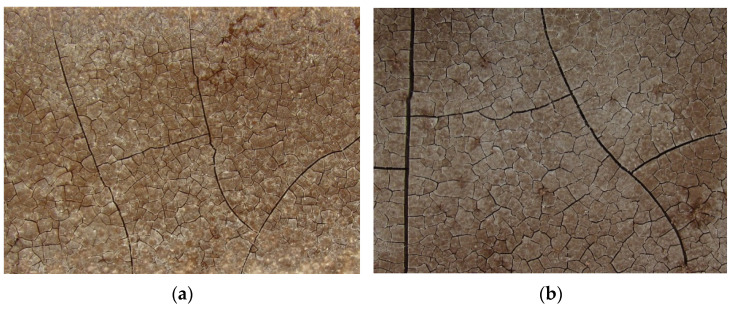
Network of cracks formed in the surface layer in the case of the samples with borax addition exceeding 5 wt.%; (**a**) MP-7.5-P and (**b**) MP-10-P.

However, it is necessary to mention that the use of a higher borax dosage, namely in the case of samples MP-7.5-P and MP-10-P, led to the separation of a thin surface layer of the manufactured samples that may have been caused by an inhomogeneity of the samples and their segregation is molds; the separation was accompanied by the crack development in the surface layer. The cracks were continuously expanding during the hardening process (see Figure 5). Such effect disrupted the material integrity. If this effect of borax addition persisted in mortars, it could cause a significant worsening of the mechanical properties. Therefore, to avoid cracking development, the use of 0–5 wt.% of borax was considered to be suitable for the use in mortars.

Based on the rheological performance, the samples MP-2.5-P and MP-5-P were considered as the potential reference borax-containing binders for the MKPC-based mortar design. The efficiency of 2.5 wt.% of borax was surprisingly high; the experiments revealed that the samples with the mentioned retarder amount can provide a slower rate of the change in viscosity, temperature, and Young’s modulus in time in comparison to the samples with a different (higher) borax amount. However, the mixture containing 5 wt.% of borax was chosen in the end, as it provided a longer setting time than MP-2.5-P, and managed to maintain a good integrity without visible defects. Additionally, the lower initial modulus of elasticity growth rate in the case of MP-2.5-P may be a disadvantage, as high initial mechanical properties are important for the materials used for repair purposes. Accordingly, taking into consideration mechanical parameters of pastes, material MP-5-P exhibited the best mechanical resistance among borax-enriched materials. For these reasons, the next part of this study uses the composition of MP-5-P and combines it with different aggregates to manufacture mortars with a wide range of desirable properties.

### 3.2. Properties of the Hardened Mortars

The results of mortar testing are summarized in the following paragraphs. Unlike the paste-focused part of this study, the mortar testing was aimed mainly at the properties of mortars in the hardened state.

The first part of mortar results, the basic material characteristics of the hardened mortars and workability of the fresh mortars, are summarized in Table 8. All the mortars containing borax addition showed higher spread diameter than the control mortar MP-REF-0. The measured spread values showed that the set W/C ratio equal to 0.25 was high for all the borax-containing mortars, as the borax application led to a significant decrease in viscosity. Such behavior was responsible for the rise in the spread values—in the case of reference mortar with 5 wt.% of borax, the borax addition caused the increase in the spread diameter value by 70 mm compared to the reference sample. This finding is in agreement with a study concluded by Yang et al. [37], who noticed that the increase in borax share enhances the MKPC paste fluidity.

Moreover, the impact of the aggregate type was observed. The full replacement of silica sand by Liaver (MP-L) even increased the obtained spread values to 250/260 ± 5 mm; such a fact may have been caused by the oval shape and smooth surface of the Liaver particles. On the contrary, the use of rubber granulate partly served as a counterweight to the borax effect, and slightly lowered the spread value to 210/220 ± 5 mm. In that case, the spread value was again affected by the aggregate shape—the used rubber aggregate was characteristic by particles with rough surface and sharp edges. In the case of the mortars containing a combination of Liaver and rubber aggregate, the spread value was almost as high as the one measured for the mortars containing Liaver only. Overall, for a practical use, it would be necessary to decide a specific fitting ratio for each aggregate type a planned application.

The basic structural properties measured for the dried samples also reported noticeable material differences in dependence on the type of used aggregate. In the case of the reference samples, both specific and bulk density of the mortar proved to be relatively high; the bulk density was slightly higher than in the case of the reference paste, and the specific density was slightly lower. The reference sample containing 5 wt.% of borax showed basically the same specific density, but a higher value of bulk density, and therefore lower total open porosity compared to the sample without borax. The same effect of borax addition was observed for the MKPC pastes in the first part of the research; the denser structure and therefore the lower porosity is likely the result of the less viscous consistency of the fresh mixture.

**Figure 6:**
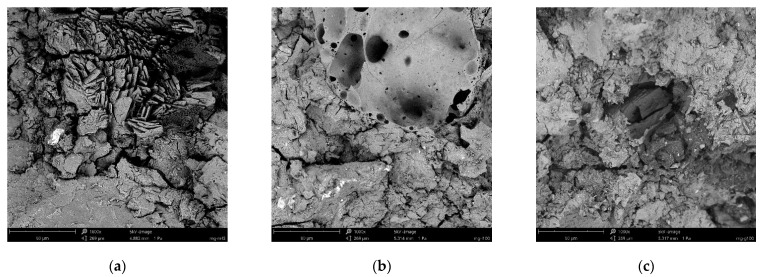
Microstructure of MKPC-based mortars gained by SEM, magnification 1000×. (**a**) MP-REF 5, various degrees of hydration; (**b**) MP-L, the transition between the magnesia-phosphate hydration product and expanded glass grain; (**c**) MP-R, non-compact hydrated structure around the rubber particle.

As for the properties of mortars containing different aggregates, those considerably depend on the parameters of the applied aggregates. The less dense and visibly non-compact structure of the used rubber granulate (see Figure 6) resulted in quite low bulk density of the final composite MP-R, but also low specific density resulting in total open porosity even lower than the one obtained for the reference sample without the borax addition. Meanwhile, the highly porous expanded glass as a sand substitution caused a very low bulk density of the Liaver-containing mortar and as a consequence that mortar also reached the highest open porosity. This feature is typical when lightweight porous aggregate is used as silica sand substitution. Based on the obtained results it is possible to claim that the Liaver incorporation outweighs the effect of borax addition in terms of total open porosity—even with a huge spread value, and the high total open porosity is achieved. This observation is supported by the previous research conducted by Pavlíková et al. [53], where the growing share of Liaver in MOC-based mortars caused the increase in open porosity.

The thermal properties measured on dried mortar samples are introduced in Table 9. Those results show that while the borax addition basically does not affect thermal conductivity of the materials, it causes a slight decrease in thermal diffusivity and an increase in volumetric heat capacity. On the other hand, the aggregate substitution affects all the measured thermal parameters. The incorporation of both crumb rubber and expanded glass aggregate and also their combination resulted in the lowering of the thermal conductivity of the mortars. The lowest thermal conductivity was measured for the sample MP-L, the highest was measured for the sample MP-R; however, both values were considerably lower than the thermal conductivity of both reference mortars. Overall, the highly porous Liaver structure enhanced the thermal behavior, and Liaver-containing mortars ended up being the best thermal insulation material of all the examined possibilities. However, the gained thermal conductivity was still high in comparison to the standard thermal insulation materials [67,68].

The thermal diffusivity values measured for samples containing Liaver and rubber were comparable and lower than the thermal diffusivity values of the reference samples. The lowest value, 0.53 m^2^·s^−1^, was measured for the MP-R composite. The heat storage ability represented by the volumetric heat capacity oscillated around 1.60 × 10^6^ J·m^−3^·K^−1^ for the samples containing rubber and Liaver, with the reported lowest value 1.29 × 10^6^ J·m^−3^·K^−1^.

The experimentally assessed heat transport and storage parameters reflect the arrangement of the mortar inner structure formed from dense magnesia-phosphate binder and aggregates with variable porosity, shape and morphology. The inverse proportionality between the calculated open porosity and measured thermal conductivity can be observed—e.g., the sample containing Liaver aggregate showed the highest open porosity and the lowest thermal conductivity. Thus, higher porosity is connected with higher thermal insulation ability and also with slower heat transport. Overall, the use of lightweight aggregates enhances the thermal insulation ability of MKPC-based mortars. A similar effect of rubber and expanded glass aggregate incorporation on thermal parameter of MOC-base mortars was also reported by Pavlíková et al. [53].

The obtained water transport parameters of the hardened MKPC-based mortars are shown in Table 10. These parameters determine the rate of water transport and storage and thus clearly affect the water resistance against liquid water penetration and the consequential water-induced damage. Except the water absorption coefficient, which was very similar for all examined mortars, the highest values of 24 h water absorption *W*_a_ and *W* were observed for the reference mortar MP-REF 0, showing that the composite containing silica sand aggregate and not using the setting retarder is the most susceptible to water-induced damage of all manufactured samples. The use of borax provided the other reference sample, MP-REF 5, with more compact structure that showed noticeably lower water absorption potential. Obviously, the borax use is advantageous in terms of water resistance improvement.

The acquired data further indicate that water transport parameters of MKPC-based mortars depend on the type of the used aggregate. The considerable differences in parameters between the samples containing different aggregates can be observed. Although the expanded glass is classified as a highly porous material which gives high porosity value of the hardened mortar, it provides the samples with moderate moisture transport ability and hygric parameters only slightly higher than received for mortar with silica sand aggregate. The high porosity is probably balanced by a non-absorbing repellent character, which partially reduced the effect of high porous inner structure. From the point of view of moderation of water ingress, the most advantageous hygric parameters were measured for the samples containing crumb rubber, which was due to its hydrophobic nature recently reported by Chen et al. [69].

Table 11 shows the mechanical parameters represented by compressive and flexural strength measured for mortars at the age of 14 days. Trends of both values are in agreement. The highest mechanical resistance (compressive strength 34.57 MPa, flexural strength 7.2 MPa) was detected in the case of the reference composite MP-REF 0. The incorporation of borax into the reference mixture caused a decrease in the 14 days flexural value, but the compressive strength remained unaffected. The drop in mechanical strength on a larger scale was observed when silica sand aggregate was replaced by expanded glass and rubber granulate. The worst mechanical properties were measured for the composite containing 100% of rubber aggregate, despite the rubber granulate samples had lower porosity compared to Liaver. This issue may be caused by an insufficient bond between the hydrated magnesia-phosphate structure and rubber particles that is likely affected by the rough surface of the used rubber aggregate; a closer look at this issue is presented in Figure 6.

**Figure 7:**
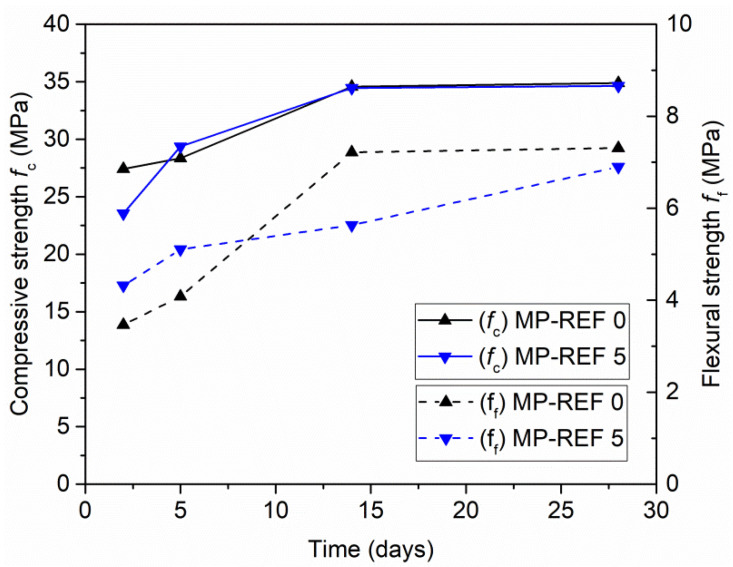
Strength development of reference mortars—age 2, 5, 14, and 28 days.

Reference mortar MP-REF 0 as well as MP-REF 5 were characterized in terms of time dependent strength development. The results (Figure 7) showed the gradual increase in strength values. The differences between the early strength values (after 2 and 5 days) were quite low. However, it should be noticed that while the higher compressive strength at the age of 2 days was observed for MP-REF 0, the higher flexural strength at the same age was provided by MP-REF 5. As for the compressive strength, at 14 days it reached the same and final value for both reference samples. Meanwhile, the flexural strength of MP-REF 0 reached the final value at 14 days and on the contrary, flexural strength of MP-REF 5 kept growing until the age of 28 days. The maximal measured flexural strengths were basically the same for both reference mortars.

## 4. Conclusions

The research was focused on the design and testing of magnesium potassium phosphate-based materials. In the first part, several magnesium phosphate fresh mixtures with varying dosage of setting retarder borax were characterized. The significant improvement of setting properties caused by the borax addition was observed. The sample containing 5 wt.% of borax was evaluated as the most advantageous mixture in terms of setting time elongation and sample homogeneity. This mixture was subsequently used as the binder for MKPC-based mortars investigated in the second part of this research. The chosen binder was supplemented by the following aggregates: quartz sand, expanded glass, and crumb rubber granulate processed from waste tires. The aggregates were chosen with respect to the current global tendency to use recycled and thermally effective materials. Highly porous expanded glass was the representative of the material with thermal insulation potential; the application of the waste rubber was chosen as it helps to process the continuously increasing amount of this waste and brings financial benefits. The observation of composites consisting of the listed materials led to the following highlighted findings:

(i) The application of borax in MKPC pastes resulted in the extension of the setting time, slower temperature development rate together with lower temperature peaks, and better workability. The longest setting time was provided by the samples containing 10 wt.% of borax.
(ii) Milder hydration reaction caused by the borax addition was accompanied by postponing of the Young’s modulus of elasticity increase.
(iii) The use of rubber and expanded glass aggregate in the MKPC-based mortars significantly improved the thermal insulation properties of the developed composites compared to the reference material containing silica aggregate only.
(iv) All tested composites containing lightweight aggregate exhibited low permeability for water, with rubber aggregate providing the best water resistance.
(v) The mechanical strength values detected for the materials containing lightweight aggregate were lower compared to those of the mortars containing silica sand aggregate. However, the gained values were still sufficient for some possible material applications; these newly developed materials could be used, e.g., as thermal insulation facing slabs, repair materials for improvement of thermal insulation performance reducing water imbibition, etc.
(vi) Compared to Portland cement-based materials, reference mortars reported very high early compressive and flexural strength at the age of 2 and 5 days, respectively.

Based on the conducted tests and analyses of the newly developed materials, it can be claimed that the magnesia-phosphate-based composites with borax addition represent a promising, low-carbon alternative to traditional Portland cement-based materials, applicable for example in the field of structural rehabilitation. Moreover, the addition of lightweight aggregates noticeably improved the thermal insulation performance of the resulting material and allowed MKPC-based materials to be more thermally effective compared to concrete.

The continuation of this research is going to focus on the incorporation of another type of retarder, namely boric acid, into the MKPC-based mortars in order to find the most advantageous setting retarder. Moreover, the impact of other types of lightweight aggregates will likely be investigated. Additionally, the durability of MKPC-based materials when subjected to high temperatures may well be investigated, as the thermal properties proved to be advantageous and such investigation would therefore be the next step in assessing the possible use of MKPC-based materials for fire protection.

## Figures and Tables

**Table 1:** Chemical composition and physical properties of MgO.

Oxide Composition	MgO	SiO_2_	CaO	Al_2_O_3_	Fe_2_O_3_	MnO	SO_3_	Powder Density	Specific Density	Specific Surface
	(wt.%)							(kg·m^−3^)	(kg·m^−3^)	(m^2^·kg^−1^)
Magnesia	76.5	1.0	3.3	5.8	12.6	0.7	0.1	1972	3591	1980

**Table 2:** Particle size distribution of MgO (µm).

	d_10_	d_50_	d_90_
Magnesia	10.2	66.2	157.9

**Table 3:** Mix proportion of MKPC pastes.

Mixture	Weight Ratios			Composition (g)			
	M/P	W/C	Borax	MgO	KH_2_PO_4_	Borax	H_2_O
MP-0-P	8.0	0.25	0.0%	1273.1	410.7	0.0	346.2
MP-2.5-P	8.0	0.25	2.5%	1273.1	410.7	34.6	346.2
MP-5-P	8.0	0.25	5.0%	1273.1	410.7	69.2	346.2
MP-7.5-P	8.0	0.25	7.5%	1273.1	410.7	96.1	346.2
MP-10-P	8.0	0.25	10.0%	1273.1	410.7	128.1	346.2

**Table 4:** The chemical composition and physical properties of silica sand, Liaver, and rubber granules.

Materials	SiO_2_	Al_2_O_3_	MgO	TiO_2_	Fe_2_O_3_	Na_2_O	CaO	K_2_O	Loose Powder Density	Compacted Powder Density
	(wt.%)								(kg·m^−3^)	(kg·m^−3^)
Silica sand	97.1	2.3	0.3	0.2	-	-	-	-	1363	1910
Liaver	70.2	2.2	2.1	0.2	0.5	13.8	9.4	0.8	305	346
Rubber									441	517

**Table 5:** Ratios and weight proportion of the developed mortar mixtures.

Mixture	Weight Ratios			Composition (g)						
	M/P	W/C	Borax	MgO	KH_2_PO_4_	Borax	Sand	Liaver	Rubber	H_2_O
MP-REF 0	8.0	0.25	0.0%	1273.1	410.7	0.0	1384.7	-	-	346.2
MP-REF 5	8.0	0.25	5.0%	1273.1	410.7	69.2	1384.7	-	-	346.2
MP-L	8.0	0.25	5.0%	1273.1	410.7	69.2	-	309.5	-	346.2
MP-L+R	8.0	0.25	5.0%	1273.1	410.7	69.2	-	154.8	224.2	346.2
MP-R	8.0	0.25	5.0%	1273.1	410.7	69.2	-	-	448.5	346.2

**Table 6:** Setting parameters of the fresh MKPC pastes.

Mixture	Initial Setting Time	Final Setting Time	Duration of Setting
	(min)	(min)	(min)
MP-0-P	9:00	11:00	2:00
MP-2.5-P	11:15	19:30	7:45
MP-5-P	13:00	21:00	9:00
MP-7.5-P	14:00	24:30	10:30
MP-10-P	17:30	31:00	13:30

**Table 7:** Basic structural characteristics of the hardened pastes.

Mixture	Bulk Density (kg·m^−3^)	Specific Density (kg·m^−3^)	Total Open Porosity (%)	Compressive Strength (MPa)	Flexural Strength (MPa)
MP-0-P	2002	3103	35.5	56.4	13.1
MP-2.5-P	1888	2996	37.0	42.9	10.8
MP-5-P	2179	2946	26.0	50.9	12.1
MP-7.5-P	1831	2786	34.3	46.8	10.9
MP-10-P	1923	2999	35.9	41.0	9.3

**Table 8:** Workability of fresh mortar mixtures and basic structural properties of the hardened mortars.

Mixture	Spread Diameter	Bulk Density	Specific Density	Total Open Porosity
	(mm)	(kg·m^−3^)	(kg·m^−3^)	(%)
MP-REF 0	160/165 ± 5	2102	2849	26.2
MP-REF 5	230/235 ± 5	2257	2846	20.7
MP-L	250/260 ± 5	1361	2561	46.9
MP-L+R	240/250 ± 5	1563	2400	34.9
MP-R	210/220 ± 5	1777	2272	21.8

**Table 9:** Thermophysical properties of the hardened mortars.

Mixture	Thermal Conductivity *λ*	Thermal Diffusivity *a*	Volumetric Heat Capacity *c_v_*
	(W·m^−1^·K^−1^)	×10^−6^ (m^2^·s^−1^)	×10^6^ (J·m^−3^·K^−1^)
MP-REF 0	1.46	0.93	1.59
MP-REF 5	1.41	0.77	1.84
MP-L	0.77	0.60	1.29
MP-L+R	0.88	0.61	1.45
MP-R	0.97	0.53	1.85

**Table 10:** Hygric parameters of the hardened mortars.

Mixture	24 h-Water Absorption *W_a_*	Water Absorption Coefficient *A_w_*	24 h Water Absorption *W*
	(%)	×10^−3^ (kg·m^−2^·s^−1/2^)	(kg·m^−2^)
MP-REF 0	9.12	1.58	3.77
MP-REF 5	6.50	1.56	3.62
MP-L	6.31	1.67	5.41
MP-L+R	4.69	1.63	4.41
MP-R	3.91	1.57	3.16

**Table 11:** Mechanical parameters of hardened mortars at the age 14 days.

Mixture	Compressive Strength *f_c_*	Flexural Strength *f_t_*
	(MPa)	(MPa)
MP-REF 0	34.57	7.22
MP-REF 5	34.45	5.63
MP-L	13.64	3.26
MP-L+R	10.31	2.92
MP-R	8.21	2.47

## Data Availability

The data presented in this study are available on request from the corresponding author. The data are not publicly available due to privacy.
